# Supplementary material for: Splice-Junction-Based Mapping of Alternative Isoforms in the Human Proteome
Source: Cell Rep. Author manuscript; Available in PMC 2020 Jan 15. (PMC6961840; doi:10.1016/j.celrep.2019.11.026)
Supplement: 2 [file NIHMS1546469-supplement-2.zip › DF1/PXD010154/Heart/Heart_32_TTN_SNCTVSVHVSEPPIFR.pdf]

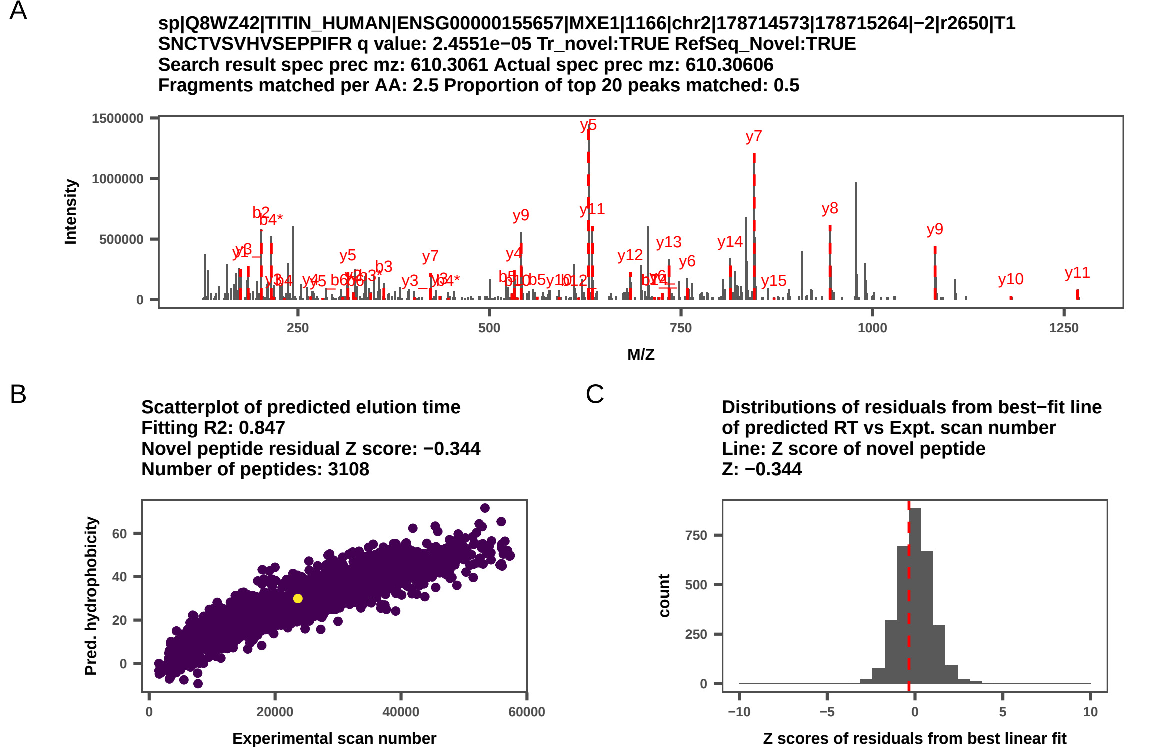
A
sp|Q8WZ42|TITIN_HUMAN|ENSG00000155657|MXE1|1166|chr2|178714573|178715264|−2|r2650|T1
SNCTVSVHVSEPPIFR q value: 2.4551e−05 Tr_novel:TRUE RefSeq_Novel:TRUE
Search result spec prec mz: 610.3061 Actual spec prec mz: 610.30606
Fragments matched per AA: 2.5 Proportion of top 20 peaks matched: 0.5
Intensity
1500000
1000000
500000
0
250
500
750
1000
1250
M/Z
B
Scatterplot of predicted elution time
Fitting R2: 0.847
Novel peptide residual Z score: −0.344
Number of peptides: 3108
Pred. hydrophobicity
60
40
20
0
0
20000
40000
60000
Experimental scan number
C
Distributions of residuals from best−fit line
of predicted RT vs Expt. scan number
Line: Z score of novel peptide
Z: −0.344
count
750
500
250
0
−10
−5
0
5
10
Z scores of residuals from best linear fit
